# Role of IFN-γ and LPS on neuron/glial co-cultures infected by *Neospora caninum*

**DOI:** 10.3389/fncel.2014.00340

**Published:** 2014-10-27

**Authors:** Erica Etelvina Viana De Jesus, Alex Barbosa Dos Santos, Catia Suse Oliveira Ribeiro, Alexandre Moraes Pinheiro, Songeli Menezes Freire, Ramon Santos El-Bachá, Silvia Lima Costa, Maria de Fatima Dias Costa

**Affiliations:** ^1^Laboratório de Neuroquímica e Biologia Celular, Instituto de Ciências da Saúde, Universidade Federal da Bahia—UFBASalvador, Brazil; ^2^Laboratório de Bioquímica e Imunologia Veterinária, Centro de Ciências Agrárias Ambientais e Biológicas, Universidade Federal do Recôncavo da BahiaCruz das Almas, Brazil; ^3^Laboratório de Imunologia e Biologia Molecular, Instituto de Ciências da Saúde, Universidade Federal da Bahia—UFBASalvador, Brazil

**Keywords:** *Neospora caninum*, neuron/glial co-culture, immune response, parasite NO downmodulation, neurite impairment

## Abstract

*Neospora caninum* causes cattle abortion and neurological symptoms in dogs. Although infection is usually asymptomatic, classical neurological symptoms of neosporosis may be associated with encephalitis. This parasite can grow in brain endothelial cells without markedly damages, but it can modulate the cellular environment to promote its survival in the brain. In previous studies, we described that IFN-γ decreased the parasite proliferation and down regulated nitric oxide (NO) production in astrocyte/microglia cultures. However, it remains unclear how glial cells respond to *N. caninum* in the presence of neurons. Therefore, we evaluated the effect of 300 IU/mL IFN-γ or 1.0 mg/mL of LPS on infected rat neuron/glial co-cultures. After 72 h of infection, LPS did not affect the mitochondrial dehydrogenase activity. However, IFN-γ decreased this parameter by 15.5 and 12.0% in uninfected and infected cells, respectively. The number of tachyzoites decreased 54.1 and 44.3% in cells stimulated with IFN-γ and LPS, respectively. Infection or LPS treatment did not change NO production. On the other hand, IFN-γ induced increased nitrite release in 55.7%, but the infection reverted this induction. IL-10 levels increased only in infected cultures (treated or not), meanwhile PGE_2_ release was improved in IFN-γ/infected or LPS/infected cells. Although IFN-γ significantly reduced the neurite length in uninfected cultures (42.64%; *p* < 0.001), this inflammatory cytokine reverted the impairment of neurite outgrowth induced by the infection (81.39%). The results suggest a neuroprotective potential response of glia to *N. caninum* infection under IFN-γ stimulus. This observation contributes to understand the immune mediated mechanisms of neosporosis in central nervous system (CNS).

## Introduction

*Neospora caninum* is a protozoan that causes cattle abortion and neurological symptoms in dogs (Wouda et al., 1998; Dubey, 1999; Jolley et al., 1999). Classical neurological symptoms are related with severe multifocal necrotizing encephalitis associated with mononuclear cell infiltration (Malaguti et al., 2012). However *N. caninum* infection is generally latent and asymptomatic, due to a formation of cysts with bradyzoites (chronic and latent parasite stage) that can be found in any organ, but is more frequently found in animal brains (Kobayashi et al., 2001; Dubey et al., 2004, 2007).

The blood-brain barrier (BBB) protects the brain against exogenous agents and is constituted by physical, metabolic, and active mechanisms (El-Bachá and Minn, 1999). However, this parasite overcomes these mechanisms and is able to infect glial cells inducing an immune regulation during protozoan infection in central nervous system (CNS) tissues (Yamane et al., 2000; Pinheiro et al., 2006a,b). Recently, Elsheikha et al. (2013) showed *that N. caninum* is able to grow in brain microvascular endothelial cells (fundamental component of the BBB) without markedly disrupting their normal proliferation or mitochondrial integrity and it was associated with an increase in infected cell respiration.

The immunopathogenesis of neosporosis is complex and only partially understood. Considering any intracellular microbial agent, cellular stress increases as the infection progresses, and host cells normally develop strategies to compensate it by metabolic shifts as an attempt to maintain energy homeostasis and cell viability, avoiding tissue damages (Elsheikha et al., 2013). Inflammatory mediators can modulate the physiology of the BBB during parasite infection, which could play important roles in CNS inflammation (Abbott, 2005). Buxton et al. (2002) and Hemphill et al. (2006) discussed that the progression of *N. caninum* infection was directed related with a balance between the tachyzoite’s ability to penetrate and multiply into host cells and the host’s ability to inhibit parasite multiplication. To clarify the pathogenesis and immune response to this parasite, some authors have studied these mechanisms in cells of the CNS. Yamane et al. (2000) observed a reduction in tachyzoite numbers in bovine cerebellar cells previously infected and stimulated with IFN-γ and TNF-α. However, the mechanism of proliferation inhibition remained unclear, except by determining that it should be independent of nitric oxide (NO) release. Despite this, the production of NO by peripheral immune response may favor the parasite penetration in the brain, since free radicals induce endothelial permeability changes (Lagrange et al., 1999).

Following these findings, Pinheiro et al. (2006a,b) proposed a rat astrocyte primary culture as a new model to study *N. caninum* infection *in vitro*. These authors observed that parasite stimulated astrogliosis and production of IL-10, TNF-α and NO. Thereafter, these authors found similar results with mixed cultures of astrocytes and microglia, observing the production of high levels of IL-6 and no detection of IFN-γ (Pinheiro et al., 2010).

*In vitro* experiments have revealed that astrocytes are necessary to establish the expression of several proteins and enzymes by brain endothelial cells (Bart et al., 2000). Therefore, infected astrocytes could interfere in the BBB function. Accurate knowledge about interactions between neuron and glial cells and *N. caninum* is required to learn how the infection can disturb the brain homeostasis. Recent studies of our research group proved that the stimulation of glial cells (astrocyte and microglia) with IFN-γ and TNF-α controlled the parasite proliferation independent of NO production, since it was synergically inhibited by IFN-γ and tachyzoites. Additionally, an increase in PGE_2_ release was observed in infected cultures, while IL-10 and TGF-β depletion seems to play a possible role on parasite persistence in infected cells. Moreover, while both regulatory cytokines did not interfere in the modulation of NO synthesis, IL-10 could stimulate the release of PGE_2_ (Jesus et al., 2013).

To continue these studies, it is necessary to understand how glial cells respond to *N. caninum* infection when neurons are present. Some studies have showed that glia can affect neurons by releasing neurotransmitters and other extracellular signaling molecules. Indeed, it is known that the interplay between resident cells of the CNS and peripheral immune response is complex and it can lead to neurotoxic or neuroprotecting effects (reviewed by Kerschensteiner et al., 2009). On the other hand, the parasite could also act in the modulation of this response. As suggested by Elsheikha et al. (2013), it is possible that the parasite could modify the cellular environment to promote its own intracellular survival.

This is necessary to understand how the interaction between *N. caninum*, neurons and glial cells affects immunopathogenic mechanisms and the response to infection. Therefore, the aim of the present study was to evaluate the effect of inflammatory stimulus (IFN-γ and LPS) on neuron/glial co-cultures infected with *N*. *caninum* in order to understand aspects of mediators release and their influence on neurotoxic/neuroprotective effects induced by the parasite infection.

## Material and methods

### Neuron/Glial co-cultures

Mixed glial cells (astrocytes and microglia) were first obtained from brain cortices of newborn rats (<48 h of age) by mechanical dissociation of the tissue. The cultures were maintained in Dulbecco’s modified Eagle’s medium-F12 (DMEM-F12) supplemented with 10% (v/v) fetal bovine serum, 100 IU/mL penicillin G, 100 g/mL streptomycin, 2 mM L-glutamine, 0.011 g/L pyruvate, 3.6 g/L Hepes and 12 mM glucose, incubated at 37°C in a humid atmosphere with 5% CO_2_. All of these reagents were purchased from Gibco/Invitrogen.

These cultures were initially seeded onto culture dishes with the diameter of 100 mm (TPP, Switzerland) and after 14 days, they were re-seeded (5 × 10^4^) in tissue culture plates with 24-wells for assays. This culture was previously characterized as containing about 86% of astrocytes and 12% of microglia (Pinheiro et al., 2010).

At this time, embryos were removed from pregnant rats on the 17th or 18th gestational day by cesarian section. Cells were dissociated from embryo brain cortices in DMEM/F-12 as described above. Neurons (2.5 × 10^4^/well) were plated on astrocyte/microglia monolayer and the cultures were maintained with regular DMEM/F-12 changed every 48 h for 7 days, when the experiments were performed. All animal procedures were performed in accordance with the local Ethical Committee for Animal Experimentation.

### Culture of *Neospora caninum*

*N. caninum* tachyzoites of the NC-1 strain were maintained in Vero cells in RPMI 1640 medium (Gibco BRL, USA) supplemented with 10% (v/v) fetal bovine serum (Gibco BRL, USA), 100 IU/mL penicillin G and 100 g/mL streptomycin (CULTILAB, Brazil). To obtain the parasites, the Vero cells were first washed with phosphate buffered saline (PBS) and then mechanically disrupted. Thereafter, tachyzoites were purified using a 5.0 µm filter (Millipore, Carrigtwohill, Ireland) as described by Pinheiro et al. (2010).

### Neuron/Glial co-culture infection and treatment

Neuron/Glial co-cultures were treated with 300 IU/mL of recombinant rat IFN-γ (R&D Systems, USA) or 1.0 mg/mL of LPS from *Escherichia coli* 0111:B4 (Sigma-Aldrich, USA) diluted in culture medium. Cells were treated only with fresh medium in control conditions. Twenty-four hours after treatment, neuron/glial co-cultures were infected with tachyzoites of *N. caninum* (host:parasite ratio of 1:1). Analyses were performed 72 h post-infection as determined in previous studies (Jesus et al., 2013).

### MTT assay

The MTT [3-(4,5-dimethylthiazol-2-yl)-2,5-diphenyl tetrazolium bromide] assay was performed to evaluate the energetic metabolic activity of cells. The assay is based in the ability of alive cells to convert yellow MTT in purple formazan crystals by mitochondrial dehydrogenases. The experiment was performed in 96-well plates (TPP, Switzerland). Briefly, cells (1 × 10^4^ cells/well) under different culture conditions were incubated with MTT at a final concentration of 1.0 mg/mL for 2 h. Thereafter, cells were lyzed with 20% (w/v) sodium dodecyl sulfate (SDS), 50% (v/v) dimethyl formamide (DMF) (pH 4.7), and plates were kept overnight at 37°C in order to dissolve formazan crystals. The optical density was quantified at 580 nm (Hansen et al., 1989). Three independent experiments were carried out with eight replicate wells for each analysis. Results are shown as mitochondrial activity percentage compared to untreated/uninfected control cultures, considered as 100%.

### Determination of parasite number

To quantify the parasite in cultures, the number of tachyzoites was counted in each culture 72 h after infection. To ensure that all parasites (intra- and extracellular) were counted, culture monolayers were scraped with their culture media, the cells were ruptured by three passages through a 22-gauge needle and tachyzoites were counted using a hemocytometer, as described by Yamane et al. (2000). Three independent experiments were performed in triplicate by two independent investigators in a blind assay. The results are expressed as the mean of tachyzoite percentages compared with the untreated control cultures (considered as 100%).

### Measurement of nitrite levels

Supernatants from neuron-glial co-cultures were assayed in triplicate for nitrite content, which reflects NO production, using the Griess reagent (1% sulfanilamide and 0.1% naphthyl-ethylenediamine dihydrochloride in 2.5% phosphoric acid in equal volumes). After 15 min of incubation at room temperature, the absorbance was measured at 560 nm using a microtiter plate reader (Biotek instruments, Inc., USA). Nitrite concentrations were calculated by comparison with a standard calibration curve of sodium nitrite (NaNO_2_:1.26–100 mM/L) with DMEM-F12 as the baseline control.

### PGE_2_ levels determination

Culture supernatants from the different treatments were assayed for PGE_2_ levels using an enzyme immunoassay kit (Cayman Chemical Co., USA), according to manufacturer instructions. This assay has a detection limit of 15 pg/mL.

### Cytokines determination

TNF-α and IL-10 were measured in the culture supernatants by using a commercial kit (Sandwich ELISA, R&D, USA), according to manufacturer instructions. Cell culture medium (three samples of three independent experiments**)** was collected 72 h after infection, centrifuged at 3500 g during 5 min and stored at −70°C until the time of assay. Results are expressed as percentage of concentration means compared to untreated control cultures, considered as 100%.

### Morphometry analysis

β-III tubulin immunocytochemistry was performed to detect neurites. Cells were incubated with mouse monoclonal anti-β-III tubulin (Santa Cruz Biotechnology Inc., USA) diluted 1:400 in TBS (tris buffer solution), overnight at 4°C in a humid chamber. Then, these cells were incubated with goat anti-mouse IgG peroxidase (1/400 in TBS, Bio-Rad, Hercules, CA) and the immunoreactivity was visualized using a peroxidase-conjugated substrate kit according to manufacturer’s instructions (Bio-Rad, Hercules, CA). Co-staining (a blue panchromic differential staining to nuclei and other cytoplasmic components) was performed by using the protocol established by Rosenfeld (1947). The Rosenfeld’s reagent (1 mL) was added and incubated for 20 min at room temperature. Thereafter, the plates were rinsed with water, air dried, analyzed and photographed in an optical phase microscope (Nikon TS-100) using a digital camera (Nikon E-4300).

Neurite lengths were determined using NIH software Image J, with Neuron J plug-in (Copyright from Erik Meijering). Three independent experiments were performed and neurites of each neuron were measured in five randomly chosen fields per sample. Results are shown as percentage of mean total neurite length compared to untreated/infected control neurons, considered as 100%.

#### Statistical analysis

The results are expressed as the mean ± the standard deviation (SD). The comparisons between the experimental groups and the corresponding controls were performed with GraphPad Prism 6 for Mac OS X (GraphPad Software, Inc.) using a two-way ANOVA, except to parasite number evaluation that one-way ANOVA followed by a Tukey post-test was performed. Probability values (p) of 0.05 or less were considered significant.

## Results

### IFN-γ decreased the mitochondrial activity

**Figure 1:**
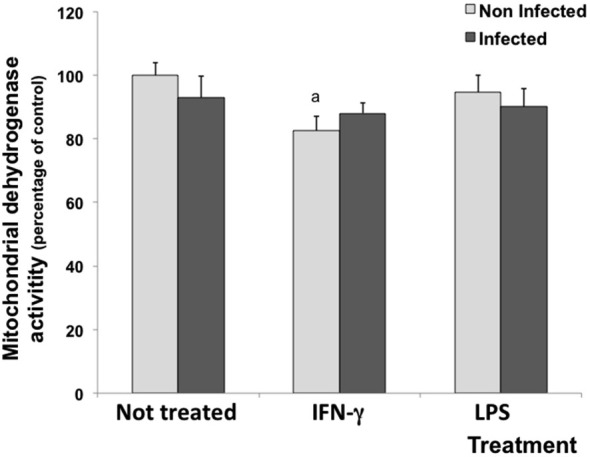
**Percentage of cell viability measured by the MTT assay in rat neuron/glial cell co-cultures treated with 300 IU/mL of IFN-γ and 1.0 mg/mL of LPS and infected with *Neospora caninum* tachyzoites (ratio cell:parasite 1:1)**. The results are expressed as the percentage of cell viability observed in different treatment conditions and the respective standard deviation compared with untreated/uninfected control cultures (considered as 100%) 72 h post infection. The results are expressed as the mean of the percentage and the respective standard of eight samples, in three independent experiments. “a” represents a significant statistical difference when compared to untreated/uninfected control cultures; (Two-way ANOVA/Tukey’s Multiple Comparison Test—*p* < 0.05).

MTT assay was performed to evaluate whether the experimental treatment with cytokines induced a metabolic challenge to cells. Under experimental conditions, untreated and LPS-treated cultures (infected or not) did not show reduction in mitochondrial dehydrogenase activities. However, IFN-γ decreased mitochondrial dehydrogenase activities by 15.5 in uninfected cells (Figure 1).

### IFN-γ and LPS decreased the parasite number

**Figure 2:**
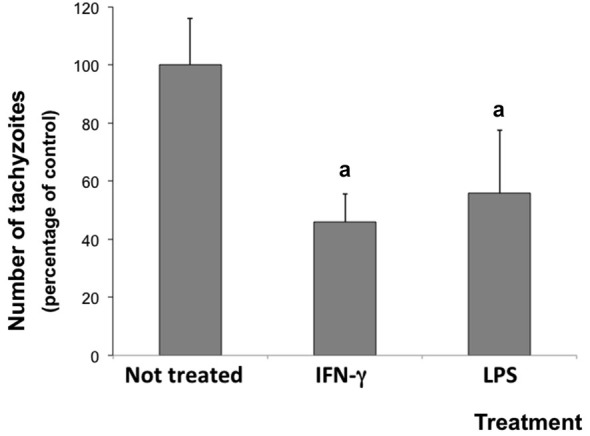
**Number of tachyzoites in rat neuron/glial cell co-cultures treated with 300 IU/mL IFN-γ or 1.0 mg/mL LPS and infected with *Neospora caninum* tachyzoites (ratio cell:parasite 1:1), 72 h post infection**. Results are expressed as means (± standard deviations) of tachyzoite percentages compared to non-treated/infected control cultures (considered as 100%) in three independent experiments carried out in triplicate. “a” represents a significant statistical difference when compared to not treated/infected cultures (One Way ANOVA/Tukey’s Multiple Comparison Test—*p* < 0.05).

The inflammatory microenvironment in neuron/glial co-cultures induced by the experimental treatment reduced the parasite number. After 72 h of infection, the number of *N. caninum* tachyzoites decreased 54.1 and 44.3% in cells stimulated with IFN-γ and LPS, respectively (Figure 2).

### IFN-γ induced NO production, but the parasite abolished this effect

**Figure 3:**
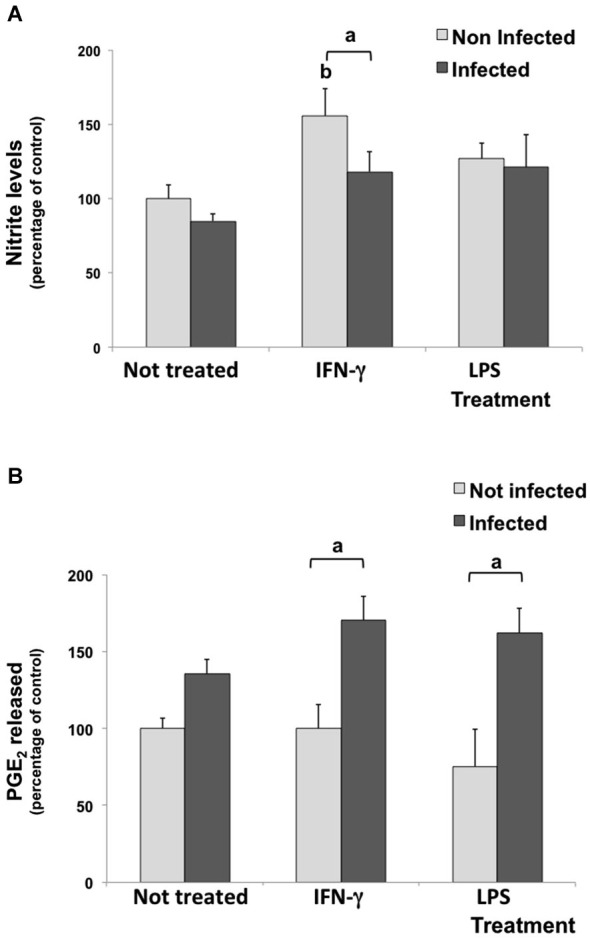
**Nitrite (A) and Prostaglandin E2 (PGE_2_) (B) levels in culture media of rat neuron/glial cell co-cultures treated with 300 IU/mL of IFN-γ and 1 mg/mL of LPS and infected with *Neospora caninum* tachyzoites (ratio cell:parasite 1:1), 72 h post infection**. Results are expressed as the means (±SD) of the percentage of nitrite **(A)** or PGE_2_ **(B)** compared to control conditions (considered as 100%) in three independent experiments carried out in triplicate. “a” represents a significant statistical difference between the same treatment group; “b” represents a significant statistical difference when compared to untreated/uninfected control cultures (Two-way ANOVA/Tukey’s Multiple Comparison Test—*p* < 0.05).

Nitrite levels were measured in culture media under inflammatory stimulus as a parameter to evaluate NO production. In untreated/infected cultures, the nitrite concentration in supernatant did not change. As expected, IFN-γ induced NO production (increased 55.7%) in uninfected cultures, but the infection decreased this induction, significantly. Meanwhile, LPS stimulus did not change nitrite levels compared with control conditions (Figure 3A).

### IFN-γ and LPS induced PGE_2_ release in infected cells

Culture media were also assayed for PGE_2_ levels. Although the inflammatory treatment with IFN-γ or LPS did not change the PGE_2_ release in uninfected cells, levels increased by 70.4% and 86.5% in IFN-γ-treated/infected and in LPS-treated/infected cultures, when compared to IFN-γ and LPS treated cultures, respectively (Figure 3B).

### LPS increased the release of TNF-α and LPS increased the release of IL-10

**Figure 4:**
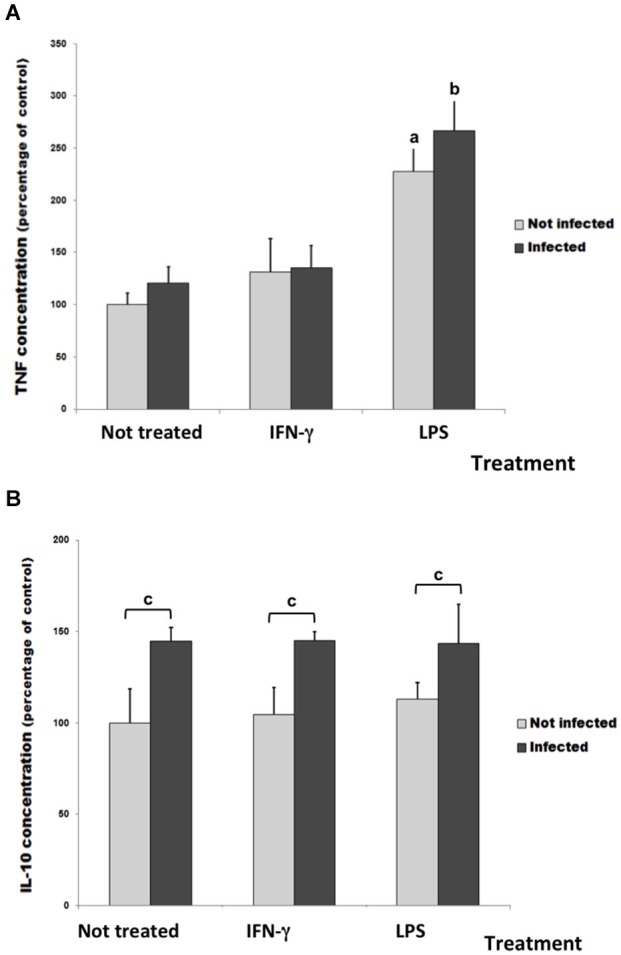
**TNF-α (A) and IL-10 levels (B) in culture medium of rat neuron/glial cell co-cultures treated with 300 IU/mL of IFN-γ or 1 mg/mL of LPS and infected with *Neospora caninum* tachyzoites (ratio cell:parasite 1:1), 72 h post infection**. Data represent the percentage of concentration means and its respective standard deviation compared to untreated control cultures (considered as 100%) three independent experiments carried out in triplicates. “a” represents a significant statistical difference when compared to untreated/uninfected control cultures; “b” represents a significant statistical difference when compared to untreated/infected cultures; “c” represents a significant statistical difference between the same treatment group; (Two-way ANOVA/Tukey’s Multiple Comparison Test—*p* < 0.05).

To investigate glial immune response in this culture infection model, the release of TNF-α and IL-10 cytokines was measured in cell culture supernatants (Figure 4). The infection or the IFN-γ treatment did not change the basal level of TNF-α measured in untreated/uninfected control cultures. However, the LPS treatment significantly increased TNF-α release both in uninfected and infected cultures (Figure 4A).

In infected cultures, the IL-10 amount in supernatant increased about 45% when compared with untreated/uninfected cultures (Figure 4B). However, IFN-γ and LPS did not change it.

### IFN-γ restored neurite outgrowth in infected cells

**Figure 5:**
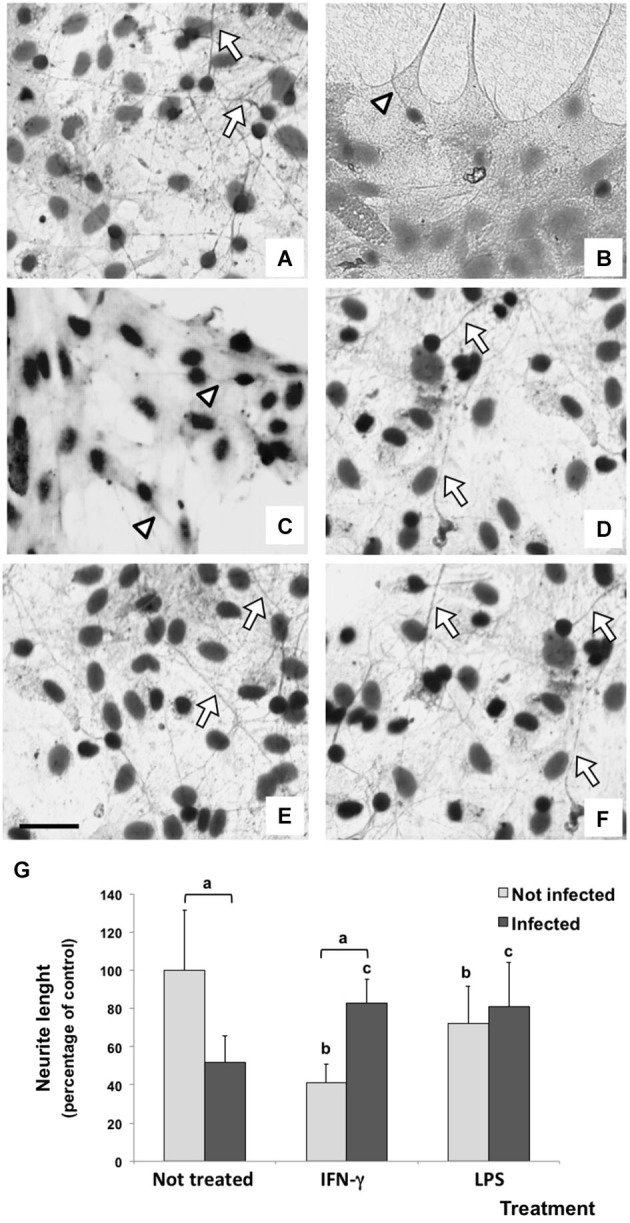
**Immunodetection (immunoperoxidase) of tubulin βIII of rat neuron/glial cells co-culture treated with 300 IU/mL of IFN-γ and 1 mg/mL of LPS and infected with *Neospora caninum* tachyzoites (ratio cell:parasite 1:1), 72 h post infection**. Control cells maintained in fresh medium exhibit a basal neurite outgrowth (arrow) **(A)**; a drastic impairment of neurite (arrowhead) was observed in *N. caninum* infected culture **(B)** and IFN-γ treated cells **(C)**; neurite length (arrow) in IFN-γ/infected cultures **(D)**; LPS/uninfected and LPS/infected cells did not exhibit changes in neurite lenght **(E,F)**. Scale bar = 50 µm. **(G)** Measurement and statistical analysis of neurite length (percentage of control) and its respective standard deviation compared to untreated/uninfected cultures (considered as 100%) in three samples in three independent experiments. “a” represents a significant statistical difference between the same treatment group; “b” represents a significant statistical difference when compared to untreated/uninfected control cultures; “c” represents a significant statistical difference when compared to untreated/infected cultures (Two-way ANOVA/Tukey’s Multiple Comparison Test—*p* < 0.05).

In this study, neurite outgrowth length was used as a parameter to evaluate the neuronal ability to maintain the dynamics of the tubulin and actin cytoskeletons (Figure 5), which is essential for the establishment of synapses. The basal neurite length under control conditions was considered as 100%. Untreated/infected neuron/glial co-cultures showed a drastic impairment of neurite outgrowth (reduction of 51.47%; *p* < 0.001), which can represent a possible deleterious effect of parasite infection. Although IFN-γ significantly reduced the neurite length in uninfected cultures (42.64%; *p* < 0.001), this inflammatory cytokine reverted the impairment of neurite outgrowth induced by the infection (81.39%).

A similar result was observed in LPS treated cultures. While neurite length reduced in LPS treated/uninfected cultures (72.3%; *p* < 0.001), LPS also inhibited a further impairment of neurite outgrowth induced by the infection (increase of 29.4% when compared with untreated/infected cultures).

## Discussion

Previous studies of our group described that astrocyte/microglia cultures infected with *N. caninum* presented a decreased parasite proliferation, release of PGE_2_ and down modulation of NO after IFN-γ stimulation (Jesus et al., 2013). These events were associated with a parasite escape mechanism and an anti-inflammatory pattern of response by infected glial cells, suggesting a possible glial protective role on nervous tissue during this parasite infection. However, the knowledge of these response effects in the presence of neurons remains unclear. In this study, we have studied the glial response to *N. caninum* infection in a neuron/glial co-culture model and their consequences.

Different from previously observed in astrocyte/microglia mixed cultures, neuron/glial co-cultures showed reduced mitochondrial dehydrogenase activities when stimulated with 300 IU/mL IFN-γ, independent of infection. Therefore, this suggests a toxic effect of this cytokine on neuron/glial co-cultures, affecting the mitochondrial function.

The BBB is more than a physical barrier constituted by tight junctions of brain endothelial cells. Enzymes involved in the detoxification of the metabolism of numerous endogenous and exogenous compounds, such as UDP-glucuronyltransferases (EC 2.4.1.17), are expressed in microsomes of the brain (El-Bachá et al., 2000), constituting a metabolic barrier. Furthermore, some studies have shown that reactive astrocytes release a wide array of mediators, including pro- and anti-inflammatory cytokines, neurotrophic or neurotoxic factors, chemokines, complement factors and reactive oxygen species (ROS; Liberto et al., 2004; Farina et al., 2007; Sofroniew and Vinters, 2010; Allaman et al., 2011). This means that there is also an immunological barrier protecting neurons against biological agents. We previously showed that *N. caninum* induced the expression of IL-10 in rat astrocyte primary cultures, but these cells did not release IFN-γ (Pinheiro et al., 2006b). However, IFN-γ is one of the most important cytokines involved in the control of *N. caninum* growth, because IFN-γ-deficient mice succumb to acute infection with tachyzoites (Nishikawa et al., 2001). Moreover, IFN-γ inhibited *N. caninum* growth in human astrocytoma cells (Spekker et al., 2009). In the present work, IFN-γ decreased the activity of mitochondrial dehydrogenases even in uninfected cells. Therefore, this may challenge neurons.

On the other hand, the inflammatory stimulus was able to reduce the parasite number in infected neuron/glial co-culture. Some previous studies have shown reduction in tachyzoite number mediated by inflammatory stimulus both *in vitro* and *in vivo* (Innes et al., 1995, 2000; Khan et al., 1997; Tanaka et al., 2000). In brain cells, Yamane et al. (2000) showed that IFN-γ and TNF-α inhibited the parasite growth in bovine cerebellar cells *in vitro*. Similarly, previous findings of our research group (Jesus et al., 2013) had already obtained a similar result in primary cultures of glial cells (astrocytes and microglia).

A large number of studies have shown the role of IFN-γ in controlling *T. gondii* proliferation, but the exact mechanism that promotes this anti-parasitic effect remains uncertain (Jun et al., 1993; Halonen et al., 1998, 2001; Halonen and Weiss, 2000; Freund et al., 2001; Scheidegger et al., 2005; Delair et al., 2009). Some evidences indicate that cytokines, mainly IFN-γ, can also activate astrocytes to inhibit the growth of* T. gondii*, but the mechanism of inhibition remains to be elucidated (Halonen et al., 1998). These authors showed that this event is not due to NO production and that the addition of tryptophan had no effect on inhibition, indicating that the mechanism was not mediated via indoleamine 2,3-dioxygenase (IDO) induction.

In the same way, studies about the role of IFN-γ in controlling *N. caninum* proliferation are controversial. Tanaka et al. (2000) indicated a NO dependent mechanism as responsible to kill tachyzoites inside macrophages. Using *in vivo* models, Baszler et al. (1999) showed the role of IFN-γ in controlling acute neosporosis in mice. However, the development of encephalitis and parasite proliferation were more related to the absence of IL-4-mediated response than a strong IFN-γ response.

The mechanisms involved on the parasite destruction induced by IFN-γ cytokine or other inflammatory stimuli still need to be clarified. Vonlaufen et al. (2002) reported that after 5 days of infection with *N*.* caninum* tachyzoites, IFN-γ treated slices of CNS organotypic cultures showed only small necrotic pseudocysts and many parasites supposedly dead after cell invasion. However, it is not yet possible to say whether the reduction in the number of tachyzoites reported in this study was due to a reduction in the invasiveness of the parasite and/or an increased ability to destroy the infected cells in their cytoplasm.

This study did not observe a reduction on nitrite levels in the supernatant of infected co-cultures. This disagrees with our previous findings, in which the infection reduced NO release in astrocyte/microglia mixed cultures (Jesus et al., 2013). However, in IFN-γ treated co-cultures, the infection reduced NO production. Rozenfeld et al. (2005) provided evidence that the NO production of IFN-γ-activated microglia is inhibited by *T. gondii* infection, which appears to favor neuron viability. Previously, Yamane et al. (2000) showed that NMMA (an iNOS selective inhibitor) did not reverse the inhibition of parasite growth by IFN-γ in cerebellar bovine cells. Therefore, we can suppose that the parasite reduction in IFN-γ-stimulated cultures might be induced through other mechanisms than NO. Nevertheless, further studies should be performed to elucidate the NO down-modulation mechanism by the parasite infection on glial cells.

Another possibility to be considered involves the consumption of NO to produce peroxynitrite anion—a potent oxidant and toxic agent—by NADPH oxidase stimulation in glial cells (Minghetti and Levi, 1998; Bal-Price et al., 2002; Brown and Bal-Price, 2003). However, peroxynitrite determination was not tested in this study.

These data suggests two interpretations: (1) the reduction in the tachyzoite number promoted by inflammatory stimuli should not be mediated by NO; (2) *N. caninum* infection in glial cells can induce parasite escape mechanisms, which could, among other things, decrease NO production. A possible direct action of the parasite is reinforced by Rozenfeld et al. (2005). These authors observed that in microglia cultures treated with IFN-γ the inhibition of iNOS expression was restricted to *T. gondii* infected cells.

Despite its classic performance as a pro-inflammatory molecule, PGE_2_ also plays a role in neuronal injury protection by decreasing NO production in activated microglia and modulating proinflammatory events (Aloisi et al., 1999; Zhang and Rivest, 2001). In *T. gondii* infection it is believed that PGE_2_ may be especially favorable to nervous tissue, modulating the immune response and contributing to maintain the integrity of brain cells (Rozenfeld et al., 2003).

In this study, we observed an increase in PGE_2_ released by IFN-γ-treated/infected and LPS-treated/infected cultures. This fact could be indirectly related with regulatory mechanisms, triggered by inflammatory stimulus, that contribute to NO down modulation in the presence of the parasite. However, further experiments should be conducted to confirm this hypothesis. PGE_2_ has been also associated with an enhancement of regulatory cytokine secretion, as IL-10. Some studies showed exogenous PGE_2_ inducing cAMP up-regulation, which leads to TNF-α and IL-12 inhibition and over expression of IL-10 (Aloisi et al., 1997, 1999; Levi et al., 1998; Rozenfeld et al., 2003).

The role of regulatory cytokine IL-10 in inflammatory and infectious diseases has been largely studied, as reviewed by Ouyang et al. (2011). This cytokine can also facilitate the tissue-healing process in injuries caused by infection or inflammation, repressing pro-inflammatory responses and limiting unnecessary tissue disruptions caused by inflammation. In this study, IL-10 was produced only in response to infection. This agrees with previous findings of (Pinheiro et al. (2006b, 2010)) who showed IL-10 overexpression by *N. caninum* infected astrocyte and in microglia/astrocyte cultures.

Due to these facts, a limited involvement of PGE_2_ and IL-10 on glial responses to infection can be supposed. However, these mediators are only two in a large number of molecules (not yet identified) that act in the complex cellular/molecular interaction during neuroglial response to *N. caninum* infection. Kerschensteiner et al. (2009) propose that the neuro-immune crosstalk in CNS is not determined by single molecules or even classes of individual molecules, but by the integration of multiple signals that individually may favor the destruction or tissue repair.

In neuron/glial co-culture, the parasite infection inhibited drastically neurite outgrowth, showing a *N. caninum*-mediated neurotoxic effect. This fact could be associated with neurological symptoms and pathological findings in infected animal (Dubey et al., 1998; Poli et al., 1998; Reichel et al., 2007; Dubey and Schares, 2011). In the same way, IFN-γ stimulus induced a severe neuronal damage. Fortunately, this cytokine was not detected in infected astrocytes and microglia cultures (Pinheiro et al., 2006b, 2010). Lipopolysaccharide induced the same inflammatory effect in neurite outgrowth. Kitayama et al. (2011) showed neurite outgrowth inhibition by LPS stimulated microglia. Indeed, a combined effect between tachyzoite and these inflammatory stimuli can be supposed, since the neurite length was preserved in IFN-γ-or LPS treated/infected cultures.

The preservation of neurite outgrowth in IFN-γ-treated/infected co-cultures can be not associated to an indirect effect of PGE_2_ and IL-10 secreted by IFN-γ-treated/infected cells as supposed by Rozenfeld et al. (2003). In this study, PGE_2_ and IL-10 were also released by untreated/not infected cultures, in which neurite impairment was more evident. A direct effect of neuron parasite infection has to be more studied to clarify how the neurotoxic effect of *N. caninum* infection occurs.

The mechanism by which IFN-γ-stimulated glia can protect neurons in *N. caninum* infected cultures are still under investigation in our research group. The IFN-γ pretreatment could induce astrocyte stored glycogen, which sustains their own energy requirements and enables them to support neighboring neurons through the export of glucose or lactate (Liberto et al., 2004). Other hypothesis is the up regulation of neurotrophic factors by glia-derived cytokines. Nerve growth factor (NGF), brain-derived neurotrophic factor (BDNF), neurotrophin-3 (NT-3) and glial cell-derived neurotrophic factor (GDNF) are able to promote neuronal survival via tyrosine kinase receptors or inhibition of NO synthetase expression (Barbacid, 1995; Wang et al., 1997).

Much more knowledge are needed to elucidate the immunoregulatory glial role during *N. caninum* infection in the CNS. The results presented here should contribute with this understanding. Under IFN-γ stimulus, the parasite reduction number associated with inhibition of NO production, release of PGE_2_ and IL-10, and neurite length preservation suggest a neuroprotective response. However, the mechanism triggered by IFN-γ stimulus and the parasite infection that leads to neuroprotection needs to be clarified. This observation can contribute to understand immune-mediated mechanisms of neosporosis in the CNS and contribute to further *in vivo* experiments. Furthermore, it contributes to understand how immunological mechanisms help to protect neurons, especially when the physical, metabolic and other mechanisms of the BBB fail to protect the brain against biological challenges.

## Conflict of interest statement

The authors declare that the research was conducted in the absence of any commercial or financial relationships that could be construed as a potential conflict of interest.
